# In This Issue

**Published:** 1994

## DIAGNOSING AND DETECTING FAS

Confirming the diagnosis of fetal alcohol syndrome (FAS) in an individual patient is often difficult, even for specialists with considerable experience, according to Dr. Jon M. Aase. Current diagnostic criteria rely on recognition of a consistent pattern of minor physical abnormalities, generalized growth retardation, and nonspecific developmental and behavioral abnormalities in children with a history of alcohol exposure before birth. Abnormalities are often subtle and hard to detect and represent the extreme of a normal spectrum of development. Though diagnostic aids may help, a definitive test for confirming prenatal alcohol damage lies in the future. (pp. 5–9)

## FAS ANIMAL STUDIES

Animal research not only has provided a model of FAS to support the findings of physical and neurobehavioral anomalies found in children with FAS, but it also has helped researchers decipher the mechanism of alcohol’s damage to the fetus. Dr. Howard C. Becker and colleagues summarize the major findings of FAS animal studies and discuss the importance of these models for future research. (pp. 10–16)

## ALCOHOL’S DISRUPTION OF CELLULAR EVENTS

Prenatal exposure to alcohol affects many crucial biochemical and cellular components of fetal development. Cellular events that may be disrupted by alcohol include cell division and proliferation, cell growth and differentiation, and the migration of maturing cells within the embryo. Drs. Elias K. and Mary L. Michaelis discuss some nutritional, hormonal, and other mechanisms by which alcohol may influence these cellular events, thereby disrupting normal organ formation, growth, and function. Knowledge of these mechanisms and events may suggest ways to help reverse or minimize some of the harmful effects of alcohol on the developing fetus. (pp. 17–21)

## CRITICAL PERIODS FOR PRENATAL ALCOHOL EXPOSURE

One focus of research on FAS has been to identify “critical periods” during gestation when the fetus is particularly vulnerable to the effects of alcohol exposure. Dr. Claire Coles reviews studies of humans and animals that attempt to associate specific FAS defects with alcohol exposure during a particular period of pregnancy. Although the formation of facial anomalies has been related to exposure early in pregnancy, Dr. Coles notes that attempts to determine critical periods for behavioral deficits have yielded less concrete results. (pp. 22–29)

## DAMAGING THRESHOLDS OF ALCOHOL

Is there a level of drinking during pregnancy—a threshold level—below which no effect is seen on the mental functions of the fetus? Drs. Joseph L. and Sandra W. Jacobson examine the complex subject of threshold using two studies that compare neurobehavioral deficits to level of prenatal alcohol exposure. The authors estimate that seven standard drinks per week may be the threshold for adverse effects on behaviors such as information-processing speed and activity level, but they emphasize that the threshold may vary by woman and by infant. (pp. 30–36)

## PATERNAL ALCOHOL INTAKE AND FETAL DEVELOPMENT

Dr. Theodore J. Cicero discusses the possible direct effects of paternal alcohol consumption on fetal development, distinguishing these effects from studies of the genetic heritability of alcoholism. The author also discusses a number of possible mechanisms whereby paternally consumed alcohol could lead to cognitive and biochemical effects. (pp. 37–41)

## TERATOGENICITY OF ALCOHOL AND OTHER DRUGS

Among women, the highest rates of alcohol, marijuana, and cocaine use occur during childbearing age. Drs. Nancy L. Day and Gale A. Richardson review the effects on the fetus of exposure to alcohol and other drugs. They discuss patterns of use and evaluate the effects of these drugs separately and in combination. The authors indicate that the drug used most frequently is alcohol followed by tobacco, marijuana, and cocaine. (pp. 42–48)

## USING MRI TO ELUCIDATE BRAIN ABNORMALITIES

Children exposed prenatally to alcohol display a spectrum of structural brain abnormalities, including smaller-than-normal brains and proportional reductions in size of specific brain areas. Sarah N. Mattson and Drs. Terry L. Jernigan and Edward P. Riley describe the use of magnetic resonance imaging (MRI) to further elucidate these abnormalities and to associate them with learning and memory deficits exhibited by some alcohol-exposed children. (pp. 49–52)

## ECONOMIC COST OF FAS

Researchers estimating the economic cost of FAS to the United States have varied widely in their results because of differences in their underlying assumptions about the disorder, such as differences in incidence rates used to make the estimates. Gregory Bloss discusses the bases for several of these estimates and notes that when considering treatment and prevention policies, the cost estimates for a single FAS case are more useful than estimates of the overall costs of the disorder nationwide. (pp. 53–54)

## NEW ASSESSMENT TOOLS FOR RISK DRINKING

Screening pregnant women for “risk drinking” is a vital step toward reducing how much they expose their fetuses to alcohol. Dr. Marcia Russell discusses the importance of screening programs and assesses the effectiveness of screening tools, focusing on brief questionnaires that are currently available to physicians and prenatal clinics. She finds that the usefulness of questionnaires in screening pregnant women has been demonstrated in some, but not all, populations. Recent studies suggest that questionnaires are more accurate if they include questions about a woman’s tolerance of alcohol, because they indirectly assess risk drinking during pregnancy. (pp. 55–61)

## FAS PREVENTION STRATEGIES

Dr. Janet R. Hankin discusses FAS prevention efforts directed at women of childbearing age. She examines the effectiveness of the alcoholic beverage warning label and the need for more active measures that target women at a local rather than national level. Although warning labels have had a slight influence on women who drink moderately, some studies show that the labels have not reduced the alcohol intake during pregnancy of women who drink heavily. Local prevention programs have direct contact with pregnant women and women of childbearing age and seem to have a more potent effect than warning labels. (pp. 62–66)

## FAS INTERVENTION STRATEGIES

Lyn Weiner and Dr. Barbara A. Morse discuss the plausibility and effectiveness of various intervention strategies that attempt to help children with FAS overcome cognitive and behavioral problems. Most of the intervention strategies discussed are based on parent and teacher observations and trial and error. However, research has begun to validate the basic principles behind these strategies, such as the need for consistency and for reduced sensory stimulation. (pp. 67–72)

## ADULTS AND ADOLESCENTS WITH FAS

In the 20 years since FAS was first diagnosed, researchers have revised their beliefs about the needs of and services for FAS patients. Dr. Ann P. Streissguth reviews studies of adolescents and adults with FAS from around the world, including followup studies of older patients in whom FAS was diagnosed at birth or during childhood. The results indicate that FAS is a lifelong disability, necessitating specific interventions and treatment. (pp. 74–81)

## TRACKING THE PREVALENCE OF FAS

Systematic surveillance of FAS is crucial to its prevention, which is a national health priority. Dr. José F. Cordero and colleagues discuss the methodological problems associated with FAS surveillance and highlight two programs that have been monitoring FAS for many years. The authors discuss the attributes of successful surveillance programs and offer suggestions to improve current and future efforts to track FAS. (pp. 82–85)

## KNOWLEDGE OF FAS

How much do certain groups in the United States know about the risks to the fetus of alcohol consumption during pregnancy? In an Epidemiologic Bulletin, Dr. Mary C. Dufour and colleagues examine data from alcohol use-related questionnaires of two National Health Interview Surveys, in 1985 and 1990. The authors look at changes since 1985 in the percentages of men and women ages 18–44 who believe that heavy drinking increases the risk of birth defects, who have heard of FAS, and who can correctly describe it. They identify the groups that show changes and also consider how the groups that are less knowledgeable about FAS could be the focus of primary prevention efforts and direct interventions that change drinking behavior. (pp. 86–92)

